# Supplementary material for: Cytokine Signaling in Pediatric Kidney Tumor Cell Lines WT-CLS1, WT-3ab and G-401
Source: Int J Mol Sci. 2024 Feb 14;25(4):2281. doi: 10.3390/ijms25042281 (PMC10889092; doi:10.3390/ijms25042281)
Supplement: Supplementary file 1 [file ijms-25-02281-s001.zip › ijms-2809901-supplementary.pdf]

International Journal of *Molecular Sciences*

MDPI

Supplementary Material to Article

Cytokine signaling in pediatric kidney tumour cell lines WT-CLS1, WT-3ab and G-401

Elizaveta Fasler-Kan [1,+,*], Milan Milošević [1,+], Sabrina Ruggiero [1,+], Nijas Aliu [2], Dietmar Cholewa [1], Frank-Martin Häcker [3,4], Gabriela Dekany [1], Andreas Bartenstein [1] and Steffen M. Berger [1,*]

[1] Department of Pediatric Surgery, Children's Hospital, Inselspital Bern, University of Bern and Department of Biomedical Research, University of Bern, Bern, Switzerland
[2] Department of Human Genetics, Inselspital Bern, University of Bern, Bern, Switzerland
[3] Department of Pediatric Surgery, Children's Hospital of Eastern Switzerland, St. Gallen, Switzerland
[4] Faculty of Medicine, University of Basel, Basel, Switzerland
[+] Equally contributed to the work
[*] Correspondence: elizaveta.fasler@insel.ch (E.F.-K.); steffen.berger@insel.ch (S.M.B.)

Immunofluorescence Analysis of Phosphorylated STAT Proteins

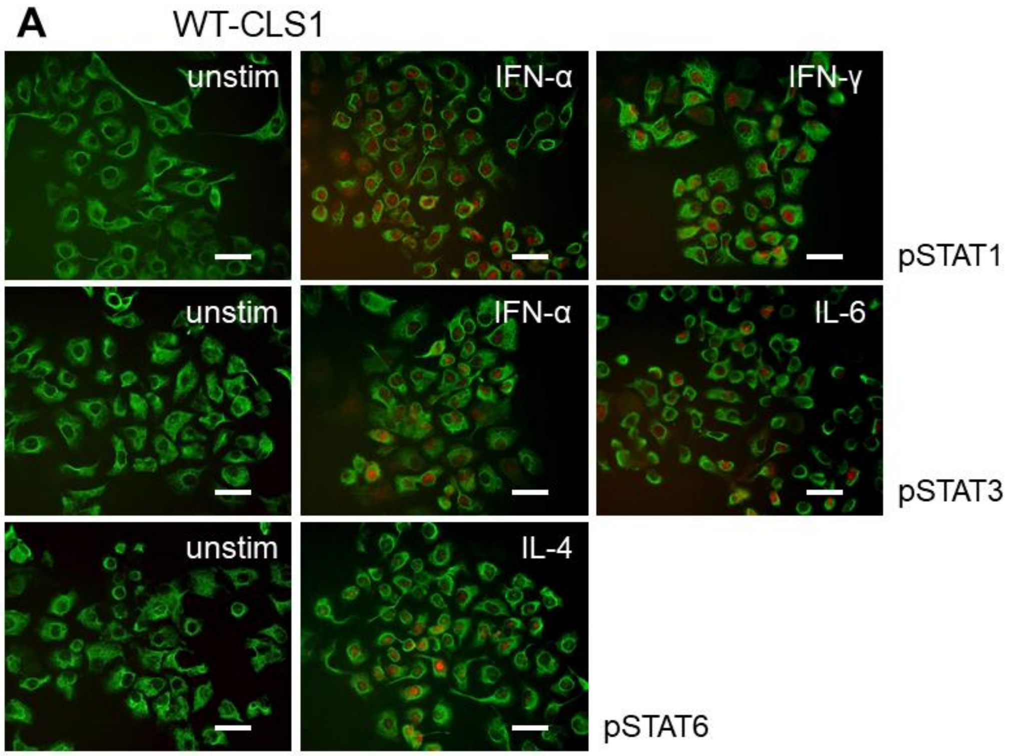

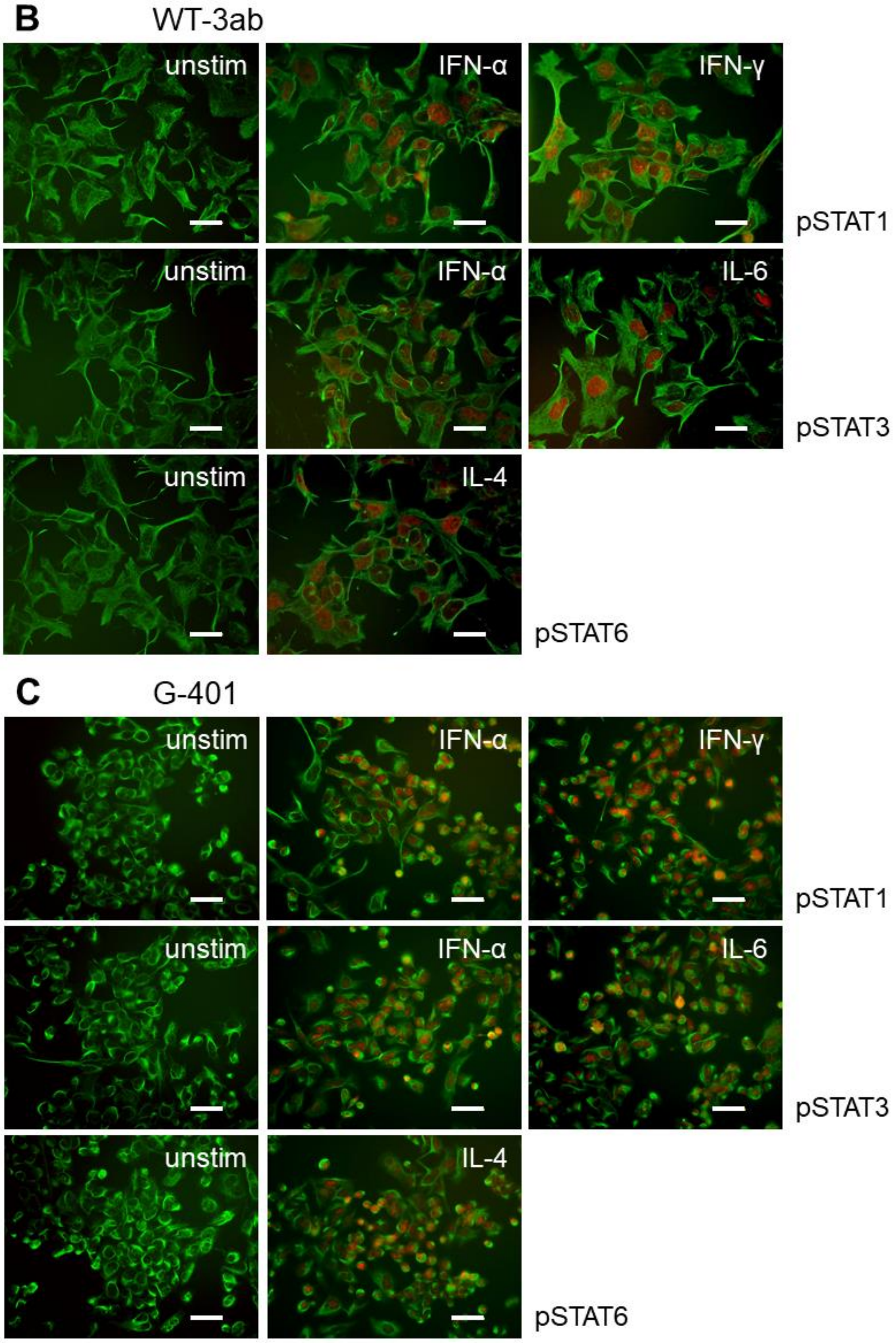

Figure S1. Immunofluorescence analysis of phospho-STAT proteins in WT-CLS1 cells (A), in WT-3ab (B) and G-401 cells (C). Cells were left untreated (left part of the panel) or treated with IFN-α (middle panel) or IFN-γ (upper right panel) and probed with anti-STAT1 (red) or anti-vimentin antibodies (green). The cells were also stained with anti-phopho-STAT3 antibodies (second row). Untreated cells served as a negative control, cells were treated with IFN-α and IL-6. On a bottom panel the cells were treated with IL-4 and probed with anti-phospho STAT6 antibodies (red). Intense nuclear staining was observed in cytokine stimulated cells. Scale bar – 50 μm.

Flow Cytometry Analysis of STAT Proteins in Response to Cytokines and MHC Modulation

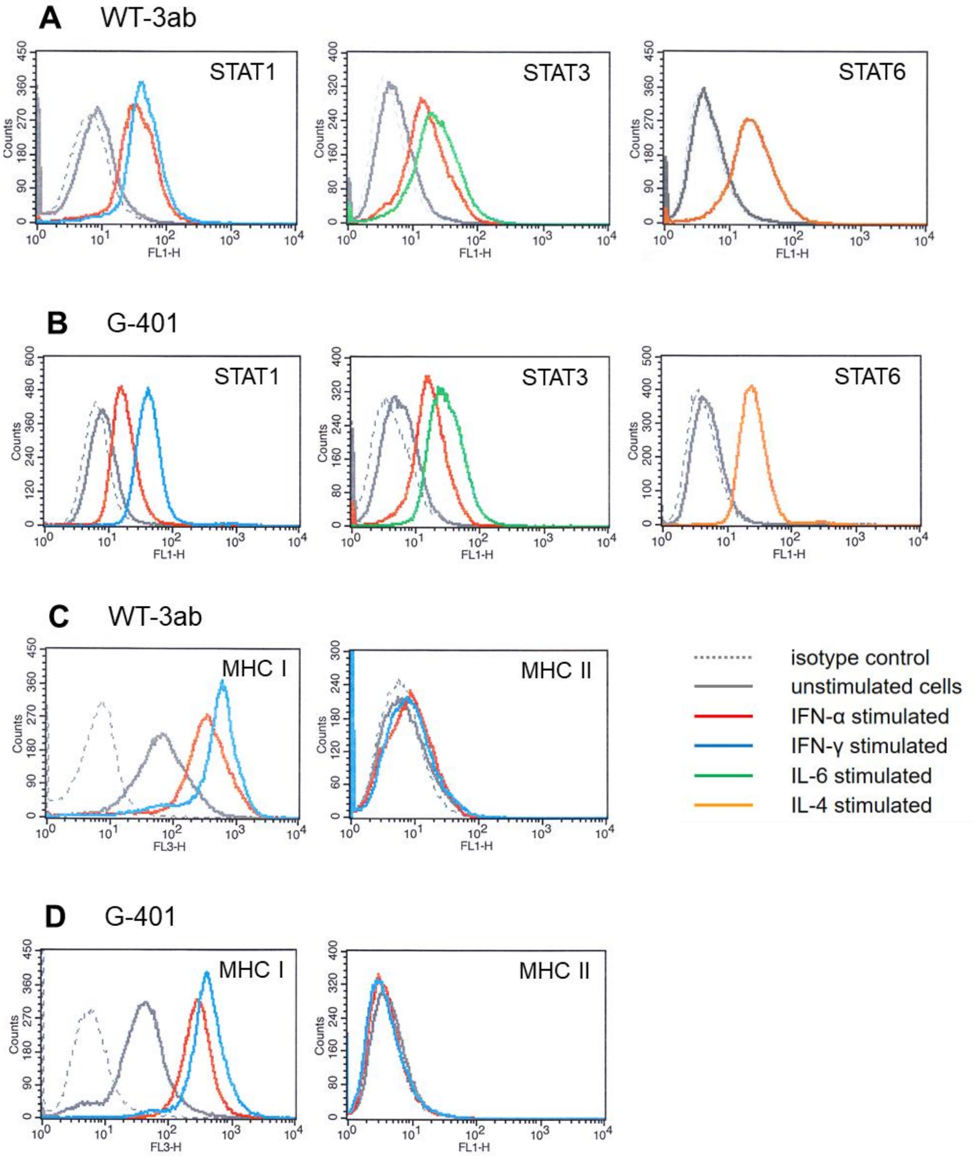

Figure S2. Flow cytometry analysis. Phospho-STAT1, STAT3 and STAT6 staining of WT-3ab (A) and G-401 cells (B) stimulated with appropriate cytokine. The fluorescence intensities of unstimulated cells (grey histograms) stained with phospho-STAT antibodies almost overlap with isotype controls (grey dotted histograms). Cells stimulated with IFN-α (red histograms) and cells stimulated with IFN-γ (blue histograms) were probed with phospho –STAT1 antibodies (left part). In the middle part are shown the histograms of STAT3 staining after stimulation with IFN-α (red histograms) and with IL-6 (green histograms). On the right part are shown the histograms of STAT6 staining after IL-4 stimulation (orange histograms).

MHC class I and class II modulation in WT-3ab (C) and G-401 cells (D). All cells express high amounts of MHC class I (grey histograms), isotype matched control are shown as grey dotted histograms. Stimulation with IFN-α (red) and IFN-γ (blue) for 72 h led to the overexpression of MHC class I molecules (figures C and D – left part). In contrast, both cell lines do not express MHC class II and stimulation with IFNs did not change the expression patterns (all histograms overlap), Figure 2C and 2D (right part).

Effect of Cytokines in MTT Assay

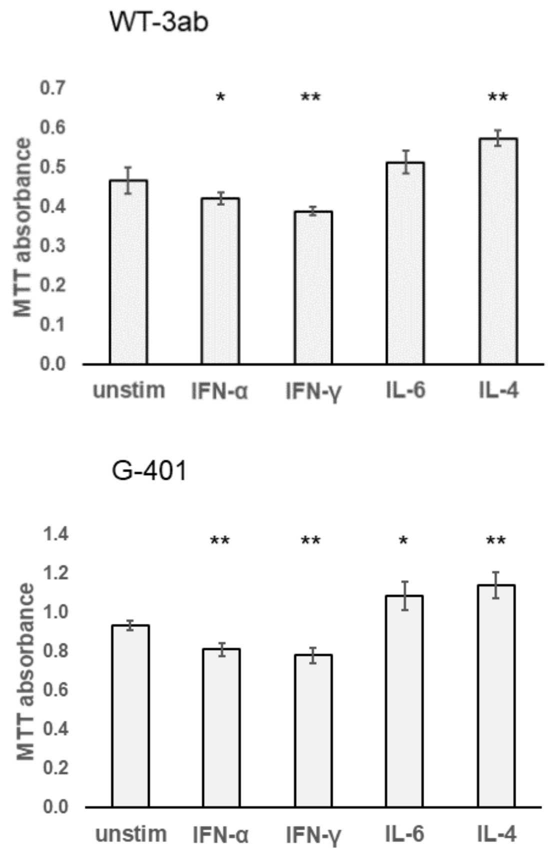

Figure S3. WT-3ab and G-401 cells were incubated with various cytokines for 96 hours. IFN-α and IFN-γ suppressed, but both IL-6 and IL-4 have increased the metabolic activity of WT-3ab (upper part) and G-401 cells (lower part). Values represent mean ± SD. Three independent experiments were performed in triplicates. Statistical probabilities (p) were expressed as * when $p<0.05$; ** when $p<0.01$.

Chromosomal Analysis

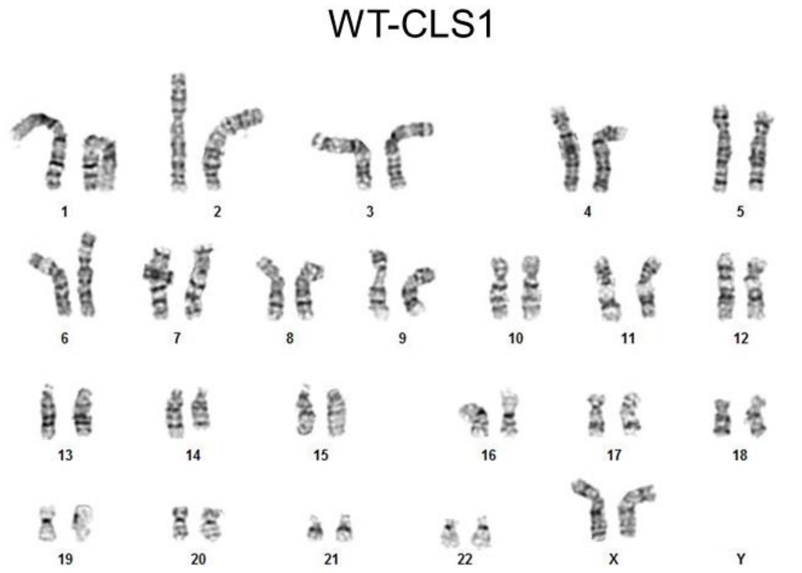

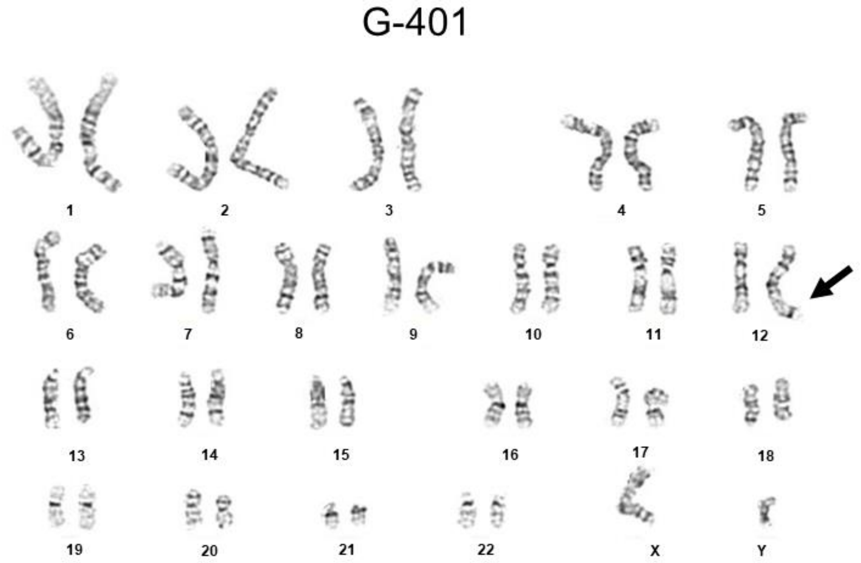

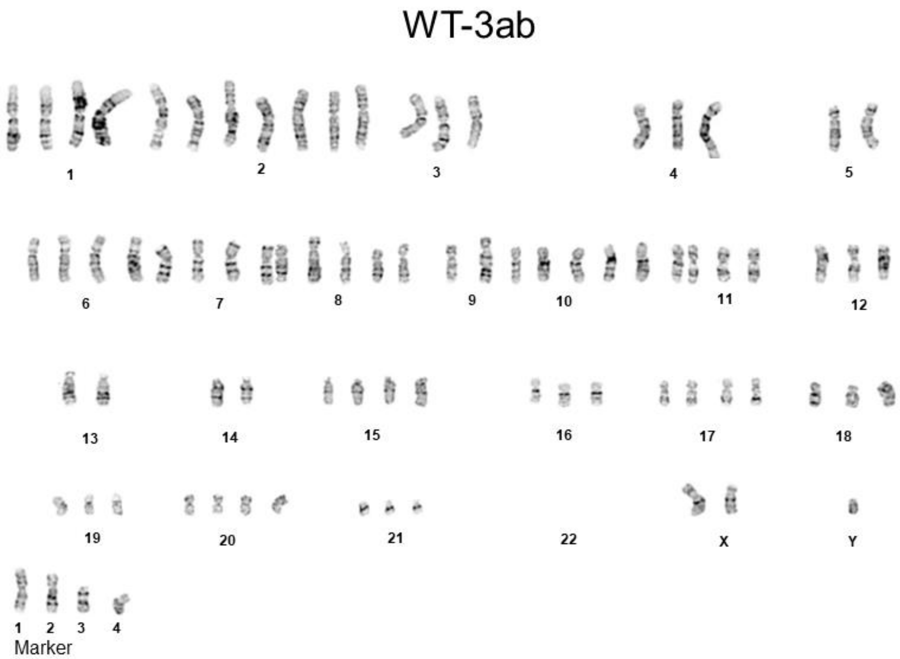

Figure S4. Chromosomal analysis (karyotyping)
WT-CLS1:
46,XX, normal female karyotype (46,XX).
G-401:
46,XY,der(12)t(7;12)(p1?4;q2?3), abberant (only structural) male karyotype with a derivative chromosome 12 (an arrow indicates this sole anomaly).
WT-3ab:
81,X,der(X),Y,+2,+2+2+2+2,+der(3),+der(4),+6,+6,+7,+7,+8,+8,+8,+9,+der(9),+10,+der(10)x2,+11,+12,+15,+15,+16,+17,+17,+der(18),+19,+20,+20,+20,+21,+22,+22,+mar1,+mar2,+mar3,+mar4,
abberant (numerical and structural) male karyotype.

Cytokine Signaling in WT-CLS1, WT-3ab and G-401 Cell Lines upon Stimulation with Interferons and Interleukins

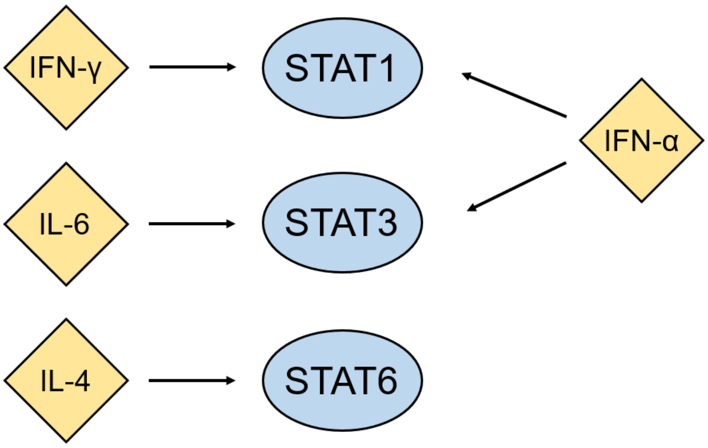

Figure S5. Scheme indicates a specific activation of STAT proteins in response to interferons (alpha and gamma) and interleukins (IL-6 and IL-4).
